# A Unique Strategy for Polyethylene Glycol/Hybrid Carbon Foam Phase Change Materials: Morphologies, Thermal Properties, and Energy Storage Behavior

**DOI:** 10.3390/ma11102011

**Published:** 2018-10-17

**Authors:** Xiaolong Su, Shikui Jia, Guowei Lv, Demei Yu

**Affiliations:** 1 Department of Chemistry, School of Science, Xi’an Jiaotong University, Xi’an 710049, China; suxl1117@stu.xjtu.edu.cn (X.S.); shikuijia@snut.edu.cn (S.J.); guoweilv@stu.xjtu.edu.cn (G.L.); 2 State Key Laboratory of Electrical Insulation and Power Equipment, Xi’an Jiaotong University, Xi’an 710049, China

**Keywords:** polyethylene glycol, hybrid carbon foam, phase change, thermal energy storage

## Abstract

Polyethylene glycol (PEG)/hybrid carbon foam (CF) phase change materials (PCMs) were prepared by integrating PEG into CF via dynamic-vacuum impregnation. The hybrid CF was first synthesized by mixtures of graphene oxide (GO) and carbon nanotubes (CNTs) with different volume ratios. The morphologies, chemical structures, thermal conductivities, shape-stabilization levels, and photo-thermal energy conversion levels of these composite PCMs were characterized systematically. The prepared composite PCMs exhibited good shape-stabilization levels and showed their original shapes without any PEG leakage. It was found that the polyethylene glycol/carbon foam with multi-walled carbon nanotubes (PEG/MCF) composite PCMs had a better shape-stable performance below the temperature of 250 °C, and the thermal conductivity of the PEG/MCF composite PCMs reached as high as 1.535 W/(mK), which was obviously higher than that of polyethylene glycol/carbon foam with single-walled carbon nanotubes (PEG/SCF, 1.159 W/(mK)). The results of the photo-thermal simulation tests showed that the composite PCMs had the ability to absorb light energy and then convert it to thermal energy, and the maximum thermal energy storage efficiency of the PEG/MCF composite PCMs and the PEG/SCF composite PCMs was 92.1% and 90.6%, respectively. It was considered that a valuable technique to produce high-performance composite PCMs was developed.

## 1. Introduction

Nowadays, the grave environmental crisis and the shortage of fossil fuels drive the development and efficient utilization of various kinds of renewable energy. Thermal energy storage, a popular method of energy storage, plays an important role in improving energy utilization by coordinating the energy supply and demand. Three kinds of thermal energy storage (TES), including sensible heat storage, latent heat storage, and thermal-chemical heat storage, are widely used at present [1,2,3]. Among these, latent thermal energy storage using phase change materials (PCMs) is one of the most widely applied in various fields, including building energy, air-conditioning, solar thermal storage, smart textiles, heat pumps, waste heat recovery, and electronic devices [4,5,6,7,8,9]. All of these applications rely on the distinguished behaviors of PCMs, including their high latent heat storage, usability at larger scales, low cost, and quasi-constant phase change temperatures.

In accordance with the phase change process, PCMs are divided into four categories: solid–solid PCMs, solid–liquid PCMs, solid–gas PCMs, and liquid–gas PCMs [10]. At present, intensive research has been conducted on solid–liquid PCMs, where polyethylene glycol (PEG) is one of the most promising solid–liquid PCMs because of its excellent properties, such as a high phase change enthalpy, good chemical properties, bio-degradation, non-toxicity, excellent resistance to corrosion, a lack of decomposition within its melting/freezing temperature range, and competitive price [11,12,13,14]. Although PEG exhibits superior properties, two major challenges impede its application in the development of PCMs; namely, its poor macroscopic stability and its poor thermal conductivity [15,16].

To overcome the problem of PEG leakage during the solid–liquid phase change process, researchers have used different materials to encapsulate PEG, such as polymer materials, metal materials, and porous materials. Among these materials, porous materials can effectively prevent PEG leakage due to their high porosity, large specific surface area, and unique pore structure. At the same time, when combined with the actual cost benefit, the method for the synthesis of porous materials and phase transition materials is very simple. Ahmet prepared three kinds of kaolin-based composite PCMs (Kb-CPCMs) with latent heat values of 27.23, 32.80, and 34.63 J/g, respectively, by means of vacuum impregnation [17]. The thermal cycle experiments showed that the thermal reliability of the Kb-CPCMs changed only slightly after 1000 heating and cooling cycles. In addition, their team prepared bentonite clay (BC) and pumice sand organic PCMs. Differential scanning calorimeter (DSC) tests showed that the melting temperature of bentonite clay phase change materials (BCPCMs) was in the range of 20~23 °C, and the latent heat was in the range of 20~55 J/g, which indicated their potential for low-temperature passive TES applications. Through the introduction of graphite, the thermal conductivity of the BCPCMs had been significantly improved and their melting time was reduced [18].

On the other hand, it is also important to solve the problem of the low thermal conductivity of PEG. The addition of high thermal conductivity materials, such as graphene, mesoporous carbon, and carbon nanotubes, can effectively improve the thermal conductivity and enhance the thermal energy storage capacity of phase change materials. The Tang group fabricated PEG-based shape-stabilized PCMs using graphene oxide (GA) with different oxidation degrees [19]. The PEG/GA composite PCMs exhibited superior shape stabilities, high energy storage densities, outstanding thermal reliabilities, and more efficient photo-to-thermal energy conversion values. Under a constant loading of 7 Newtons, the dimension of PGA2-40 retained 90.3% of its original dimension after being heated from 35 °C to 150 °C. Qian used single-walled carbon nanotubes and PEG in a special customized impregnation method to synthesize nanoscale composite PCMs that exhibited a high adsorption of PEG (as high as 98%) and could still maintain their original shapes with no leakage of PEG even after 400 cycles [20].

Carbon aerogels are used to prepare supporting frames for PCMs due to their high adsorption and porosity. Recently, Yang prepared PCMs of PEG with a cellulose/GNP frame and the experimental results showed that they had good shape stability and high thermal conductivity [21]. Previous studies have shown that a carbon aerogel is very suitable as a support system for composite PCMs because it can provide a suitable sealing structure to control the leakage of composite PCMs. To develop PCMs with both high thermal conductivity and an energy conversion property simultaneously, a hybrid carbon foam (CF) of graphene oxide (GO) and carbon nanotubes (CNTs) was prepared and used as a supporting frame for composite PCMs of PEG in this work. GO plays the role of a skeleton to control liquid leakage during the phase change, and the CNTs serve as a bridge to improve the thermal conductivity of the composite PCMs. The microstructure, crystalline phase, thermal energy storage, and energy conversion of each of the composite PCMs were also evaluated in detail.

## 2. Experimental

### 2.1. Materials

PEG (*M*_n_ = 10,000 g/mol, analytical reagent) was obtained from Aladdin Reagent (Shanghai, China). GO (average thickness of sheet: 1 nm, average diameter of sheet: 0.2~10 μm), multi-walled carbon nanotubes (MWCNTs, average length: 10~30 μm, average diameter: 8 nm), and single-walled carbon nanotubes (SWCNTs, average length: 5~20 μm, average diameter: 1~2 nm) were supplied by Suzhou Tanfeng Graphene Technology Co., Ltd. (Suzhou, Jiangsu, China).

### 2.2. Synthesis of Hybrid Carbon Foam

Typically, a MWCNTs (or SWCNTs) aqueous dispersion (5 mg/mL) was added to a GO aqueous dispersion (5 mg/mL) with a volume ratio of GO:MWCNTs (or SWCNTs) = 1:0.3, 1:0.5, 1:0.7, 1:0.9, or 1:1.1, respectively. The GO-MWCNTs (or SWCNTs) aqueous mixtures were prepared under an ultrasonication treatment for 1 h and subsequent magnetic stirring for 1 h at room temperature. The aqueous mixtures were freeze-dried under vacuum at −20 °C by a vacuum freeze dryer. The obtained samples were the hybrid carbon foam with different volume ratios of carbon nanotubes and GO, and were marked as MCF-0.3, MCF-0.5, MCF-0.7, MCF-0.9, and MCF-1.1 (or SCF-0.3, SCF-0.5, SCF-0.7, SCF-0.9, and SCF-1.1), respectively.

### 2.3. Fabrication of Composite PCMs

**Figure 1:**
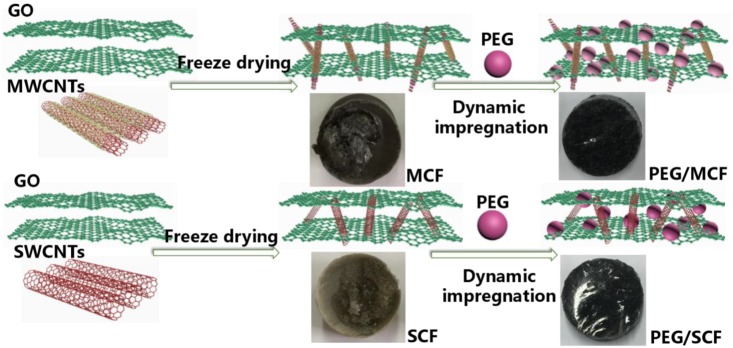
Schematic of the preparation of the hybrid carbon foam and the composite phase change materials (PCMs). GO, graphene oxide; PEG, polyethylene glycol; MCF, carbon foam with multi-walled carbon nanotubes; SCF, carbon foam with single-walled carbon nanotubes; MWCNTs, multi-walled carbon nanotubes; SWCNTs, single-walled carbon nanotubes.

The composite PCMs were prepared by the dynamic-vacuum impregnation method. This method utilizes a continuous and synchronous ultrasonic vibration and vacuum field, which can accelerate the dip rate of the PEG and enhance the impregnation effect. PEG was first melted at 90 °C in a beaker. After melting completely, the CFs with different volume ratios were added to the PEG melt with ultrasonic vibration and absolutely impregnated with the PEG melt under a vacuum condition with a pressure of 0.05 MPa. Finally, the samples were cooled at room temperature. The obtained composite PCMs were named PEG/MCF_0.3–1.1_ (or PEG/SCF_0.3–1.1_), and the mechanism is shown in Figure 1.

### 2.4. Experimental Method

The morphologies and microstructures were characterized via a field-emission transmission electron microscopy (FE-TEM) instrument (JEM-F200, Japan electronic plant type association, Tokyo, Japan) and a field-emission scanning electron microscopy (FE-SEM) instrument (Gemini 500, Carl Zeiss AG, Brunswick, Germany). The fourier transform infrared (FT-IR) spectroscopy results for the composite PCMs were recorded on a Nicolet iS50 FT-IR spectrometer (Nicolet, Madison, WI, USA) in the wavenumber range of 400–4000 cm^−1^. The X-ray diffraction (XRD) patterns of the pure PEG and composite PCMs were obtained using a D8 diffractometer (Bruker D8 Advance, Dresdon, Germany) in the range of diffraction angle 2*θ* = 5–50°, with a scan speed of 3°/min at room temperature. The Brunauer–Emmett–Teller (BET) surface area was measured using the nitrogen adsorption method at the temperature of liquid nitrogen on a Micromeritics ASAP 2020 Plus HD88 system (Micromeritics Instrument Corp, Atlantic, GA, USA). A thermogravimetry analysis (TG) was performed using a TGA-2 (Berne, Switzerland) thermal analyzer with a heating rate of 10 °C/min from 30 to 600 °C in a nitrogen atmosphere. The samples were maintained at 10 mg. The degree of crystallization was investigated using a polarizing microscope (Axioskop40, Olympus, Tokyo, Japan) with a magnification of 200× or 500×, and the samples were placed on a hot plate with a heating rate/cooling rate of 5 °C/min from 30 to 70 °C. The thermal energy storage of the pure PEG and the composite PCMs was tested using a differential scanning calorimeter (DSC, Q100, Berne, Switzerland) with a heating/cooling rate of 10 °C/min in a purified nitrogen. The samples were maintained at 10 mg to ensure instrument sensitivity. The thermal energy conversion was determined by a xenon lamp (CEL-HXUV300, Chengdu, China) and an optical power meter (CEL-NP2000, Chengdu, China). To characterize the shape-stabilization, samples of 3 × 1 cm (radius × height) in size were placed on a hot plate and digital photos of them were obtained at different temperatures. The thermal conductivity was detected with a thermal conductivity analyzer (DRH-300, Nanjing, China) using the double heat plate method. A sample of 3 × 1 cm (radius × height) in size was placed on the double hot plate, the temperature was adjusted to 30 °C, and the thermal conductivity value was recorded. Then, the temperature was adjusted to 40, 50, 60, and 70 °C, respectively, and the thermal conductivity value was recorded. The data was obtained from the average value of three measurements for each sample. Meanwhile, the shape of the samples after the measurement under different temperature was observed. The solar-to-thermal energy conversion experiment was carried out as follows. First, the intensity of a xenon lamp was adjusted to 100 mW/cm^2^ as the simulated solar light source, and then samples of 2 × 1 × 0.1 cm in size were maintained for 600 s with light, after which the light was immediately removed. During this process, the temperature change of each sample was recorded.

## 3. Results and Discussion

### 3.1. Physical Properties and Micro-Morphology of Hybrid Carbon Foam

**Figure 2:**
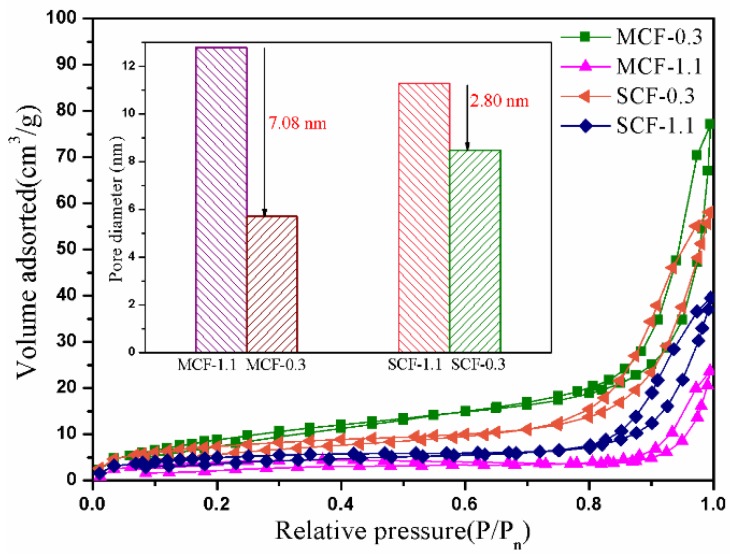
Adsorption–desorption curves of MCF-0.3, MCF-1.1, SCF-0.3 and SCF-1.1.

It is clear that the physical properties of a hybrid carbon foam, such as its pore size and adsorption quality, directly determine the PEG injection behavior in CF and then the thermal energy storage of composite PCMs. The N_2_ adsorption analysis results for the hybrid carbon foam with different MWCNTs (or SWCNTs)/GO volume ratios are shown in Figure 2. All of adsorption hysteresis loops are between 0.8 and 1.0 P/P_0_, which can be classified as type I CF [22]. As the MWCNTs (or SWCNTs) content increases, the MCF absorption capacity decreases from 77.14 to 23.58 cm^3^/g, while that for SCF is found to decrease from 58.09 to 39.48 cm^3^/g, indicating that the introduction of CNTs affects the adsorption quantity of the carbon foam. The embedded figure in Figure 2 shows that the average nano-pores size of the carbon foam “cell walls” decreases from 12.79 to 5.71 nm or 11.28 to 8.48 nm, respectively, with an increase in the nanotube content. Meanwhile, the average micro-pores size of the carbon foams with different volume ratios is 19.43~27.19 μm. This indicates that nanotubes can contribute to the formation of the carbon foam framework. The abundant pores in carbon foam play the role of PEG adsorption.

**Figure 3:**
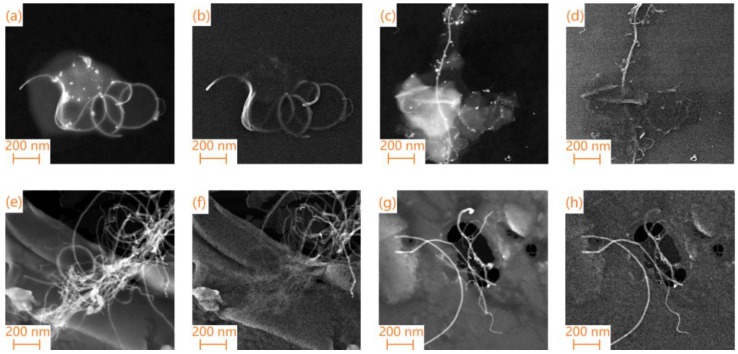
FE-TEM images of (**a**,**b**) MCF-0.3; (**c**,**d**) SCF-0.3; (**e**,**f**) MCF-1.1; and (**g**,**h**) SCF-1.1.

The morphologies and microstructures of the hybrid carbon foams with the different MWCNTs (or SWCNTs)/GO volume ratios were observed using the FE-TEM equipment. As illustrated in Figure 3a,b,e,f, it was found that the MWCNTs (or SWCNTs), with lengths ranging from several hundred nanometers to tens of micrometers, were dispersed on or between the GO nanosheets. However, it can be obviously found that excessive introduction of CNTs resulted in its accumulation on GO nanosheets, as shown in Figure 3e,f. This could be harmful to the PEG dipping process.

### 3.2. Chemical Structures of PEG and Microstructures of Composite PCMs

**Figure 4:**
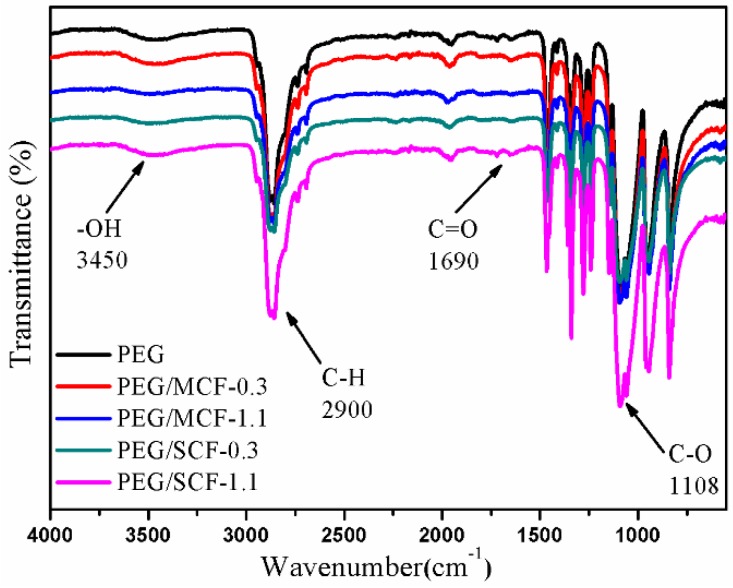
FT-IR spectrum of pure PEG and composite PCMs.

FT-IR was used to detect the chemical structures of PEG both pure and in the composite PCMs. As shown in Figure 4, the characteristic peak of pure PEG is characterized by the stretching vibration of O-H at approximately 3450 cm^−1^, the C-H stretching vibration at approximately 2900 cm^−1^, the C=O stretching vibration at 1690 cm^−1^, and the C-O stretching vibration at 1108 cm^−1^ [23]. Meanwhile, the absorption peaks of the main functional groups of PEG in the composite PCMs are almost the same as those of pure PEG, with only slight displacements. Furthermore, it was found that there were no obvious peak displacements of functional groups of PEG in the CF with a different composition and microstructure.

The microstructures of the hybrid carbon foams with different MWCNTs (or SWCNTs)/GO volume ratios and their composite PCMs were observed by FE-SEM.

**Figure 5:**
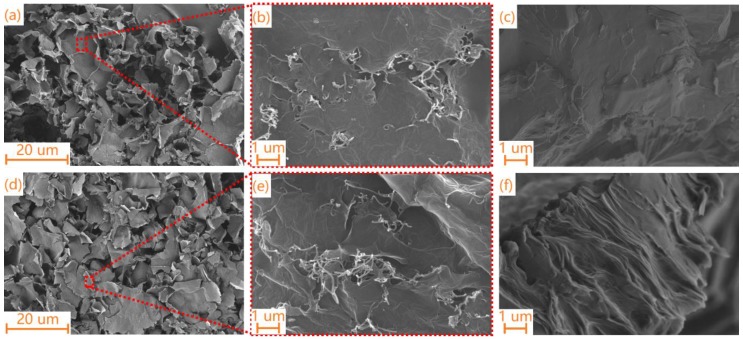
SEM images of (**a**,**b**) MCF-0.3; (**d**,**e**) SCF-0.3; (**c**) PEG/MCF-0.3 composite PCMs; and (**f**) PEG/SCF-0.3 composite PCMs.

As shown in Figure 5a,b,d,e, a porous structure of the carbon foams is found. The GO presents a sheet-like wrinkled surface, which can act as a platform to enhance the ligation of sheets with the CNTs and improve the structural stability of the hybrid carbon foam. The MWCNTs (or SWCNTs) were well-dispersed on or between the GO sheets and effectively combined with the GO sheets. The MWCNTs (or SWCNTs) played a bridge role for the entire hybrid carbon foam, effectively connecting the sheet-like structure of GO, which had a positive impact on the preparation of the composite PCMs. Therefore, the unique structure of the hybrid carbon foam was constructed by the interaction of the sheet-like GO and MWCNTs (or SWCNTs), which will improve the shape stability of the composite PCMs. As shown in Figure 5c,f, the hybrid carbon foam was filled with PEG after the impregnation. There was no terrible surface fracture or cracking in the cross-sections of the composite PCMs. This indicated that the PEG and hybrid carbon foam had good compatibility.

### 3.3. Macroscopic Phase Change of PEG and Composite PCMs

The macroscopic phase changes of the composite PCMs are very important for preventing the leakage of materials.

The shape-stable properties of the pure PEG and the composite PCMs were observed in leakage tests using a hot plate and a digital camera. The pure PEG and the composite PCMs samples were heated from 70 °C to 100 °C and maintained for 10 min with a step increase of 10 °C.

**Figure 6:**
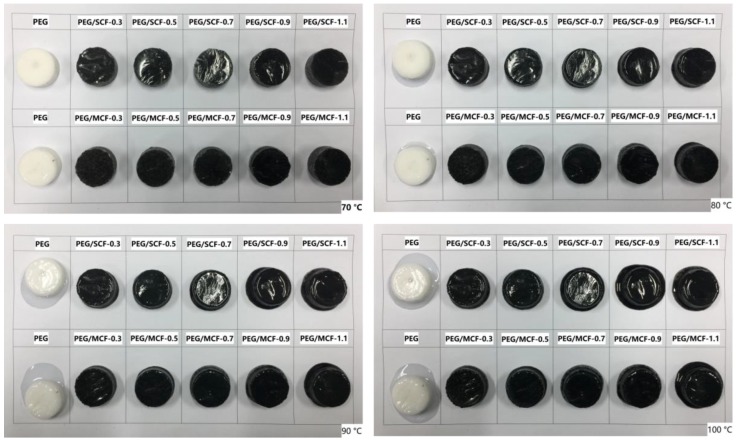
The shape-stability levels of the PEG and the composite PCMs with increasing temperature.

As shown in Figure 6, the pure PEG began to melt gradually at 70 °C, and its solid–liquid phase was formed with an increase in temperature, which caused leakage in the pure PEG sample. In contrast, the shape-stable composite PCMs were maintained at a temperature above 90 °C. PEG/SCF-1.1 and PEG/MCF-1.1 started to melt, but the leakage could not wet the filter paper at 80 °C, while the other composite PCMs retained their original shapes. When heated to 90 °C and held there for 10 min, PEG/SCF-1.1 and PEG/MCF-1.1 presented small amounts of leaked liquid, while PEG/SCF-0.9 and PEG/MCF-0.9 remained stable, with only a little melting on the bottom. When the temperature reached 100 °C for 10 min, only a little melt was found for PEG/SCF-0.7 at such a high temperature. Additionally, PEG/SCF-0.5, PEG/SCF-0.3, PEG/MCF-0.5, PEG/MCF-0.3, and PEG/MCF-0.7 did not melt throughout the heating process as a result of the unique structure of the hybrid carbon foam, which provided enough micropores to facilitate the PEG impregnation and effectively prevent the leakage of PEG. It is worth noting that the shape of the PEG/MCF composite PCMs were more stable than those of the PEG/SCF composite PCMs, which could have resulted from the mechanical property of the multiple layers of MWCNTs being more stable than SWCNTs. Thus, the phase change materials could retain a steady morphology under a relatively high temperature and prevent the PEG from escaping through encapsulation.

### 3.4. Crystallization Behavior of the PEG and the Composite PCMs

**Figure 7:**
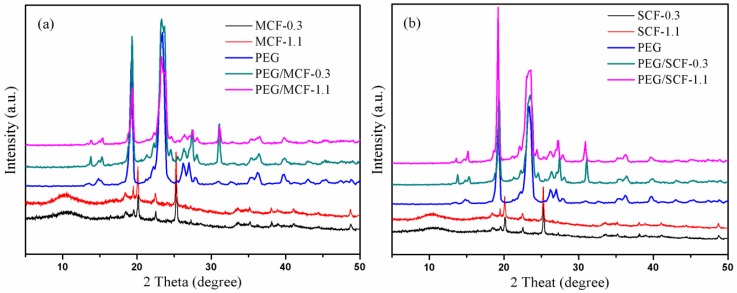
XRD patterns of (**a**) pure PEG and the PEG/MCF composite PCMs; and (**b**) pure PEG and the PEG/SCF composite PCMs.

To discuss the effects of CF on the morphology of the composite PCMs, the corresponding characterization was carried out. The structures of the MCF (or SCF) with different MWCNTs (or SWCNTs)/GO volume ratios, pure PEG, and the composite PCMs were characterized by XRD, as shown in Figure 7.

The typical diffraction peaks of the GO and CNTs were observed at approximately 2*θ* = 10.30°, 21.03°, and 25.50°, respectively, while those of pure PEG appeared at 2*θ* = 19.30°, 23.30°, 26.20°, and 27.20°, respectively [24,25]. The characteristic diffraction peak of the PEG that was in the PCMs was presented, while the characteristic diffraction peaks of the GO and MWCNTs (or SWCNTs) disappeared. PEG can completely package these MWCNTs (or SWCNTs) in the MCF (SCF). When the PEG/MCF (PEG/SCF) composites are performed by XRD, only the PEG diffraction peaks can be seen. Notably, the PEG diffraction peaks at around 2*θ* = 31.50° are visible, suggesting that MWCNTs (SWCNTs) can induce PEG crystallization. As can be seen from Figure 7a, an increase in the content of MWCNTs causes a less sharp characteristic diffraction peak of the PEG/MCF composite PCMs. This phenomenon can be explained by considering the MWCNTs as an impurity that has entered the PEG and hindered the growth of the PEG crystal itself. Compared with the MWCNTs, the characteristic diffraction peak of the PEG/SCF composite PCMs becomes more pointed with an increase in the loading of SWCNTs. This indicates that SWCNTs, as a nucleating agent, enhance the crystallization ability of PEG.

**Figure 8:**
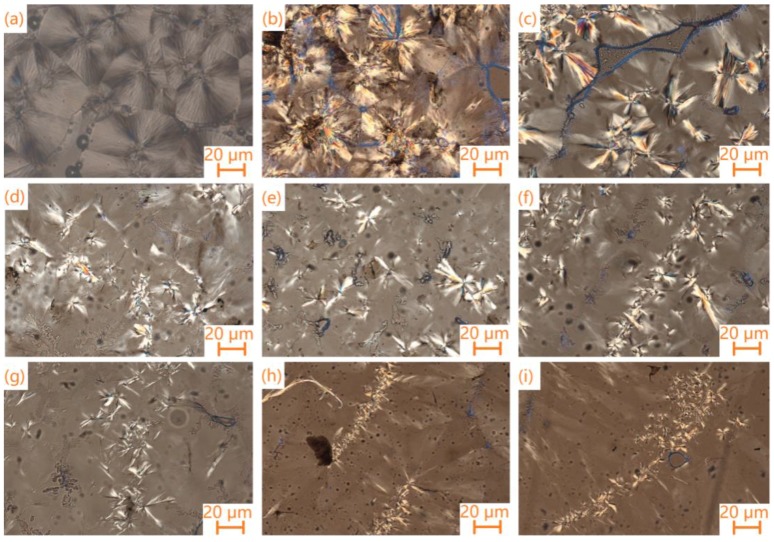
Polarizing microscope (POM) photographs of (**a**) pure PEG; (**b**) PEG/MCF-0.3; (**c**) PEG/SCF-0.3; (**d**) PEG/MCF-0.5; (**e**) PEG/SCF-0.5; (**f**) PEG/MCF-0.9; (**g**) PEG/SCF-0.9; (**h**) PEG/MCF-1.1; and (**i**) PEG/SCF-1.1 composite PCMs.

A polarizing microscope (POM) was another method used to characterize the crystallization of the composite PCMs. POM photographs of the pure PEG and the composite PCMs are shown in Figure 8.

These images show that the change in the crystalline behavior of PEG is affected by the change in the volume ratio of carbon nanotubes and GO. It is observed that the spherulite morphology of the pure PEG has a unique black cross-light pattern and uniform distribution (Figure 8a). Compared with pure PEG, when the content of MWCNTs (or SWCNTs) increases, the crystallization shape of the PEG/MCF (or SCF) composite is smaller and more fragmented until it becomes a string of small spherulite crystals. This phenomenon shows that the CNTs and GO, as a kind of impurity and/or nucleating agent, destroy the spherulite morphological characteristics of PEG, reduce the PEG crystallization rate within the limited space, and limit the molecular diffusion motion and conformational change in the molecules during crystal growth.

### 3.5. Thermal Energy Storage of the PEG and the Composite PCMs

**Figure 9:**
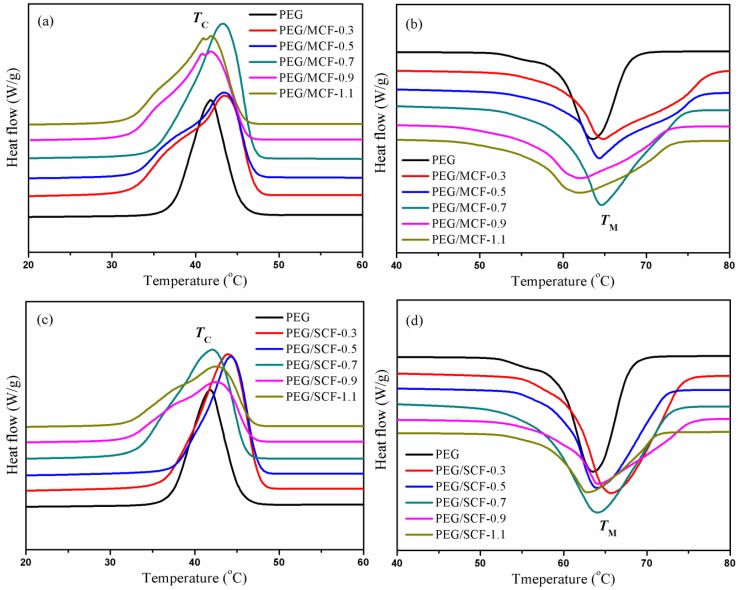
A DSC cooling scan (**a**,**c**) of pure PEG and the composite PCMs, and a DSC heating scan (**b**,**d**) of pure PEG and the composite PCMs.

By means of the DSC test method, the phase change temperature and latent heat values of the pure PEG and the composite PCMs were tested, and their ability to store heat energy was investigated as shown in Figure 9. The results of a data analysis of the initial melting/crystallization temperature (*T*_ms_/*T*_cs_), end melting/crystallization temperature (*T*_me_/*T*_ce_), peak melting/crystallization temperature (*T*_mp_/*T*_cp_), and melting/crystallization enthalpy (Δ*H*_m_/Δ*H*_c_) are listed in Table 1. The results of a data analysis of 150 cycles are listed in Table 2. The degree of crystallinity of the composite PCMs is listed in Table 3.

The endothermic peak of the composite PCMs is about 64 °C, and the exothermic peak is about 42 °C, which is very similar to the endothermic/exothermic peak range of PEG. As shown in Table 1, compared with pure PEG, the melting and crystallizing temperature regions of the composite PCMs are obviously expanded. It is considered that the GO and CNTs act as a nucleator to promote the crystallization of PEG and provide a thermal conduction pathway in the composite PCMs to accelerate the phase change process. The DSC test results show that the endothermic (Δ*H*_m_ = 135.16~155.09 J/g) and exothermic (Δ*H*_c_ = 131.38~149.83 J/g) function values of the composite PCMs are slightly lower than those of the pure PEG (Δ*H*_m_ = 173.60 J/g, Δ*H*_c_ = 160.97 J/g). The Δ*H*_m_/Δ*H*_c_ values of the composite PCMs gradually decrease with an increase in the CNTs content because an excessive amount of nanotubes could destroy the molecular structure of PEG, which further impacts the re-crystallization and melting of the composite PCMs. It can be observed from Table 2 that the measured values are almost unchanged after 150 cycles of cooling and heating. This proves that the composite PCMs have good thermal cycling stability.

As a key parameter of the composite PCMs, the degree of crystallinity is closely related to the thermal energy storage. The value of degree of crystallinity *X* can be obtained from Equation (1):

(1) $$X=\frac{\Delta H_{m}}{\Delta H_{0}w}\times 100\%$$

where Δ*H*_0_ is the melting enthalpy value of pure PEG when it is tested by DSC, and Δ*H*_0_ = 173.6 J/g, *w* is the content of PEG in the composite PCMs.

As can be seen from Table 3, the degree of crystallinity of the composite PCMs showed a gradual decline with the increase of the content of carbon nanotubes. This is because carbon nanotubes, as a kind of impurity and/or nucleating agent, limit the molecular diffusion motion and conformational change in the molecules during crystal growth, and reduce the PEG crystallization rate within the limited space.

**Figure 10:**
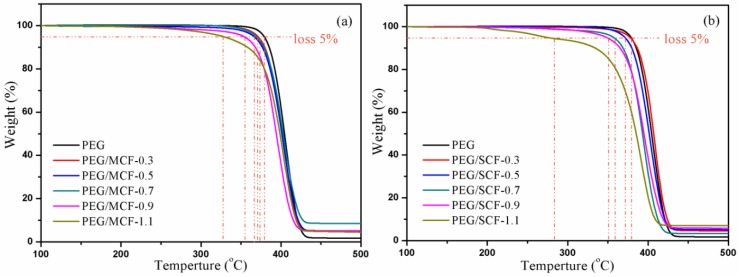
TG curves of (**a**) pure PEG and the PEG/MCF composite PCMs; and (**b**) pure PEG and the PEG/SCF composite PCMs.

The thermal stability levels of pure PEG and the composite PCMs were characterized using TG as shown in Figure 10.

As can be seen from Figure 10a, the PEG/MCF composite PCMs have maintained a good thermal stability level with almost no mass loss under 250 °C, which shows that the PEG/MCF composite PCMs have high thermal stability levels in the range of the phase change temperature and can be used in energy storage. However, it can be seen from Figure 10b that the thermal stability levels of the PEG/SCF composite PCMs are worse than those of the PEG/MCF composite PCMs. This is because the unique interlayer structure of MWCNTs can enhance stability of CF.

The mass loss of the composite PCMs is over 90% from Table 4. It is illustrated that CF provides a huge network space that increases the amount of PEG injection. So, the leakage of PEG of the composite PCMs is prevented and the thermal stability of the PCMs is improved.

Thermal conductivity is also relevant to energy storage applications because it directly affects the energy absorption and energy conversion.

**Figure 11:**
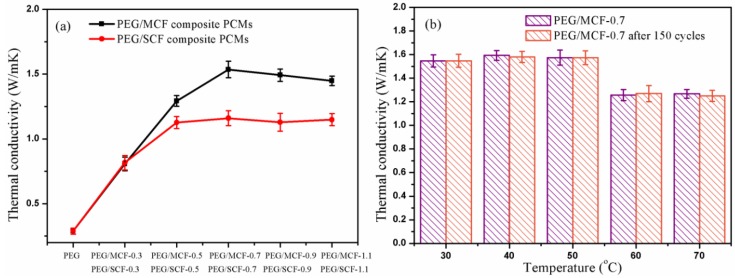
Thermal conductivities of (**a**) pure PEG and the PEG/MCF and PEG/SCF composite PCMs; and (**b**) PEG/MCF-0.7 composite PCMs after 150 cycles.

As shown in Figure 11, the thermal conductivity of pure PEG is 0.289 W/(mK), while the thermal conductivities of the PEG/MCF composite PCMs and the PEG/SCF composite PCMs reached as high as 1.535 W/(mK) and 1.159 W/(mK), respectively, and the mean error of thermal conductivity measurements is 4.43% and 5.97%, respectively, which indicates that the introduction of a hybrid carbon foam is very effective in enhancing the thermal conductivity. It is clear that the network, by connecting the MWCNTs (or SWCNTs) and the GO nanosheets to each other, improves the thermal conductivity of the composite PCMs. The thermal conduction in the PEG/MCF and the PEG/SCF was five and four times that in the pure PEG, respectively. It can be understood that multiple layers of MWCNTs could more efficiently bridge the oxidized graphene, and this thermal conduction bridge could effectively promote the thermal conduction property of the material. The PEG/MCF-0.7 composite PCM was chosen to investigate the thermal conduction at various temperatures after 150 cycles because it has the highest thermal conductivity and the greatest shape stability among the PCMs prepared. It is interesting that there is almost no change that takes place on the thermal conduction property of the composite PCM, and the original shape of the PCMs remains unchanged without PEG leakage, indicating that the PCMs have steady thermal conduction performance, as shown in Figure 11b.

**Figure 12:**
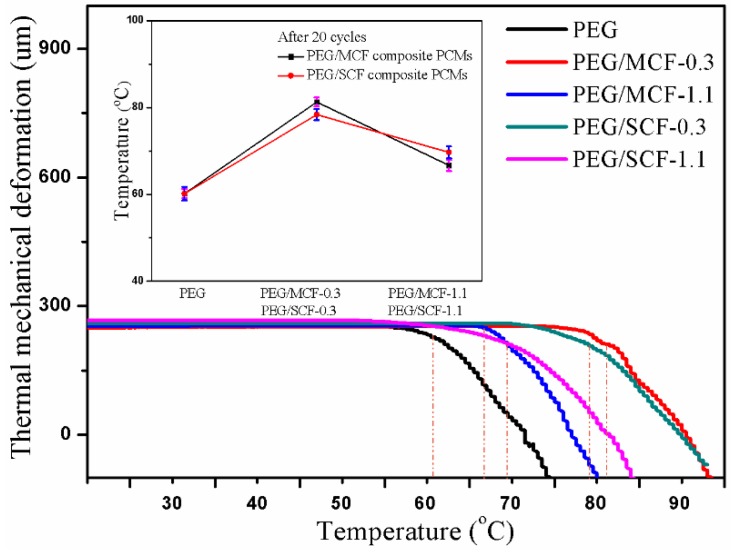
The thermomechanical analysis of PEG, the PEG/MCF composite PCMs, and the PEG/SCF composite PCMs.

The dimensional stability of the composite PCMs was tested by a thermomechanical analyzer. It can be seen from the Figure 12 that there is an inflection point in the pure PEG at 60.2 °C, while there is an inflection point in the PEG/MCF-0.3 and PEG/SCF-0.3 at 81.3 °C and 78.4 °C, respectively. It can be explained that carbon foam encapsulation of PEG can effectively improve the dimensional stability of the composite PCMs. However, as the content of carbon nanotubes increases, the deformation temperature of the phase change material decreases. This is because the excessive introduction of CNTs destroys the frame structure of CF and limits the adsorption of PEG. Thus, the deformation temperature of the composite PCMs will gradually decline. Subsequently, the samples were subjected to a thermal mechanical cycle analysis 20 times, which further proved that the composite PCMs have a good dimensional stability.

### 3.6. Thermal Energy Conversion

In order to detect whether the composite PCMs had energy storage and conversion behaviors, a simulation of a solar-to-thermal energy conversion experiment for the storage and release of the composite PCMs was conducted. Samples with a size of 2 × 1 × 0.1 cm were maintained for 600 s under a simulated solar light source with the intensity of the xenon lamp at 100 mW/cm^2^.

**Figure 13:**
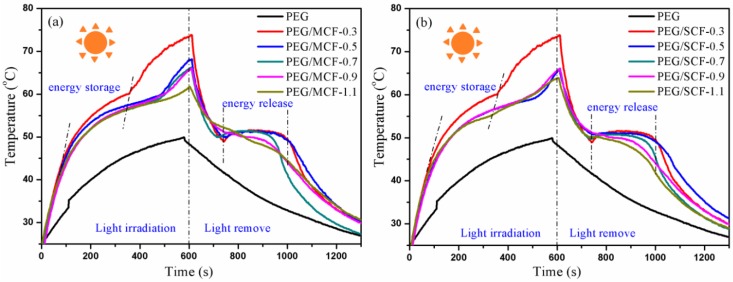
The temperature change curves of (**a**) pure PEG and the PEG/MCF composite PCMs; and (**b**) pure PEG and the PEG/SCF composite PCMs.

As shown in Figure 13, during the entire optical radiation process, the composite PCMs absorbed rays. This was followed by an increase in temperature until the inflection point appeared (at about 80~360 s), which indicated that the heat storage effects of the composite materials had already occurred. When the light source was removed, the temperature of the composite PCMs began to drop until a large platform emerged, indicating that the composite PCMs were releasing energy in the process of recrystallization. The thermal energy storage efficiency (*η*) was estimated by the light radiation energy that was absorbed during the phase change. The data results can be calculated by the following equation [26]:

(2) η=mΔHPS(ts−te)

where *m* is the mass of the PCMs material, Δ*H* is the enthalpy value of phase change, *P* is the intensity of the simulated light source, *S* is the surface area of the sample, and *t*_s_ and *t*_e_ are the starting and ending time of phase change, respectively.

By calculation, the maximum thermal energy storage efficiency of the PEG/MCF composite PCMs and the PEG/SCF composite PCMs was 91.2% and 90.6%, respectively. The results are significantly better than those of Yang et al. [27]. Moreover, the thermal energy storage efficiency of the PEG/MCF composite PCMs is higher than that of the PEG/SCF composite PCMs. The explanation for this is that the interlayer structure of MWCNTs in the composite PCMs affects its energy storage as well as its thermal conductivity and morphological stability.

## 4. Conclusions

In this study, composite PCMs, such as PEG/MCF (or PEG/SCF), with outstanding shape-stabilization, good thermal conductivity, and superior energy conversion, simultaneously, were prepared by introducing a hybrid CF. The hybrid carbon foams were fabricated by freeze-drying of a mixture of GO and MWCNTs (or SWCNTs), which showed a unique structure to enhance the impregnation of PEG and prevent its leakage in composite PCMs. It was found that there is no leakage of the melted PEG at 100 °C for a series of composite PCMs, including PEG/MCF-0.3, PEG/MCF-0.5, PEG/MCF-0.7, PEG/SCF-0.3, and PEG/SCF-0.5. It was indicated that the PEG/MCF composite PCMs had better thermal stability below 250 °C, and the thermal conductivities of the PEG/MCF composite PCMs reached as high as 1.535 W/Mk, which was obviously higher than that of PEG/SCF. The results of the photo-thermal simulation tests showed that the composite PCMs with different CNTs had the ability to absorb light energy and convert it to heat energy. The maximum thermal energy storage efficiency of the PEG/MCF composite PCMs and the PEG/SCF composite PCMs was 91.2% and 90.6%, respectively, which has potential application in energy storage.

## Figures and Tables

**Table 1:** Heating and cooling characteristics of pure PEG and the composite PCMs.

Samples	Melting Process				Solidifying Process			
	Δ*H*_m_ (J/g)	*T*_mp_ (°C)	*T*_ms_ (°C)	*T*_me_ (°C)	Δ*H*_c_ (J/g)	*T*_cp_ (°C)	*T*_cs_ (°C)	*T*_ce_ (°C)
PEG	173.6	63.90	53.34	69.77	160.97	42.29	47.34	36.48
PEG/MCF-0.3	153.3	66.54	53.26	77.58	145.08	43.96	48.35	32.37
PEG/MCF-0.5	144.6	64.05	54.23	75.39	140.34	43.81	48.66	32.03
PEG/MCF-0.7	142.7	64.63	52.34	73.22	139.34	44.53	48.57	35.32
PEG/MCF-0.9	135.7	62.88	53.87	70.97	133.15	42.34	47.54	31.89
PEG/MCF-1.1	135.1	62.74	53.15	71.40	131.38	42.36	47.39	32.15
PEG/SCF-0.3	155.0	66.14	54.11	74.83	149.83	44.18	48.60	37.83
PEG/SCF-0.5	142.5	64.25	53.29	72.47	140.48	44.49	48.29	36.41
PEG/SCF-0.7	140.2	64.15	54.75	73.53	139.20	42.38	47.51	32.06
PEG/SCF-0.9	138.5	64.08	53.05	71.44	138.08	42.21	47.48	32.11
PEG/SCF-1.1	135.8	63.13	54.33	75.03	133.98	42.01	47.53	31.27

**Table 2:** Heating and cooling characteristics of the composite PCMs after 150 cycles.

Samples	Melting Process				Solidifying Process			
	Δ*H*_m_ (J/g)	*T*_mp_ (°C)	*T*_ms_ (°C)	*T*_me_ (°C)	Δ*H*_c_ (J/g)	*T*_cp_ (°C)	*T*_cs_ (°C)	*T*_ce_ (°C)
PEG/MCF-0.3	152.5	64.44	51.32	75.49	143.12	42.89	46.42	31.32
PEG/MCF-0.5	143.2	62.20	53.53	73.42	138.40	41.90	47.61	30.15
PEG/MCF-0.7	141.6	62.58	50.28	72.22	138.38	43.48	47.60	33.30
PEG/MCF-0.9	134.1	61.48	52.54	69.87	131.17	40.39	45.57	29.75
PEG/MCF-1.1	134.8	60.82	51.17	69.46	130.32	41.41	45.23	30.38
PEG/SCF-0.3	154.1	64.35	52.21	72.76	147.79	42.20	46.67	35.91
PEG/SCF-0.5	141.8	62.13	51.23	70.51	138.54	42.53	47.30	35.52
PEG/SCF-0.7	138.4	63.18	52.71	71.54	137.37	41.27	46.45	30.08
PEG/SCF-0.9	136.2	62.11	52.09	70.50	136.21	41.28	45.51	31.17
PEG/SCF-1.1	133.9	61.21	52.21	73.10	131.29	40.14	45.48	30.29

**Table 3:** The degree of crystallinity of the composite PCMs.

Samples		Samples	
PEG/MCF-0.3	92.2%	PEG/SCF-0.3	94.1%
PEG/MCF-0.5	88.0%	PEG/SCF-0.5	87.4%
PEG/MCF-0.7	89.8%	PEG/SCF-0.7	83.6%
PEG/MCF-0.9	83.5%	PEG/SCF-0.9	85.3%
PEG/MCF-1.1	81.8%	PEG/SCF-1.1	84.8%

**Table 4:** TG data for PEG and the PEG/MCF and PEG/SCF composite PCMs.

Samples	Mass Loss (%)	*T* at Loss 5% (°C)	*T*_int_ (°C)	*T*_end_ (°C)
PEG	98.66	382.4	358.7	435.2
PEG/MCF-0.3	95.75	375.6	350.5	433.7
PEG/MCF-0.5	94.64	371.3	344.9	434.4
PEG/MCF-0.7	91.48	366.8	334.3.	424.6
PEG/MCF-0.9	94.12	358.4	318.3	420.9
PEG/MCF-1.1	95.05	334.6	288.7	422.1
PEG/SCF-0.3	94.88	377.2	353.6	433.5
PEG/SCF-0.5	93.96	369.6	355.3	431.6
PEG/SCF-0.7	96.55	357.4	328.5	423.7
PEG/SCF-0.9	93.53	351.3	329.1	425.3
PEG/SCF-1.1	92.15	284.4	214.9	411.5

*T*_int_ and *T*_end_ represent the temperatures where decomposition begins and ends, respectively.

## References

[1] Arce M.E., Alvarez Feijoo M.A., Suarez Garcia A., Luhrs C.C. (2018). Novel Formulations of Phase Change Materials-Epoxy Composites for Thermal Energy Storage. Materials.

[2] Maxa J., Novikov A., Nowottnick M. (2018). Thermal Peak Management Using Organic Phase Change Materials for Latent Heat Storage in Electronic Applications. Materials.

[3] Zhang D., Chen M., Liu Q., Wan J., Hu J. (2018). Preparation and Thermal Properties of Molecular-Bridged Expanded Graphite/Polyethylene Glycol Composite Phase Change Materials for Building Energy Conservation. Materials.

[4] Qi L., Pan H., Zhu X., Zhang X., Salman W., Zhang Z., Li L., Zhu M., Yuan Y., Xiang B. (2017). A portable solar-powered air-cooling system based on phase-change materials for a vehicle cabin. Energy Convers. Manag..

[5] Wang Y., Tang B., Zhang S. (2013). Single-Walled Carbon Nanotube/Phase Change Material Composites: Sunlight-Driven, Reversible, Form-Stable Phase Transitions for Solar Thermal Energy Storage. Adv. Funct. Mater..

[6] Sarier N., Onder E. (2012). Organic phase change materials and their textile applications: An overview. Thermochim. Acta.

[7] Marin P., Saffari M., de Gracia A., Zhu X., Farid M.M., Cabeza L.F., Ushak S. (2016). Energy savings due to the use of PCM for relocatable lightweight buildings passive heating and cooling in different weather conditions. Energy Build..

[8] Anisur M.R., Mahfuz M.H., Kibria M.A., Saidur R., Metselaar I.H.S.C., Mahlia T.M.I. (2013). Curbing global warming with phase change materials for energy storage. Renew. Sustain. Energy Rev..

[9] Liu H., Wei Z., He W., Zhao J. (2017). Thermal issues about Li-ion batteries and recent progress in battery thermal management systems: A review. Energy Convers. Manag..

[10] Chandel S.S., Agarwal T. (2017). Review of current state of research on energy storage, toxicity, health hazards and commercialization of phase changing materials. Renew. Sustain. Energy Rev..

[11] Qi G.-Q., Liang C.-L., Bao R.-Y., Liu Z.-Y., Yang W., Xie B.-H., Yang M.-B. (2014). Polyethylene glycol based shape-stabilized phase change material for thermal energy storage with ultra-low content of graphene oxide. Sol. Energy Mater. Sol. Cells.

[12] Alkan C., Guenther E., Hiebler S., Himpel M. (2012). Complexing blends of polyacrylic acid-polyethylene glycol and poly(ethylene-co-acrylic acid)-polyethylene glycol as shape stabilized phase change materials. Energy Convers. Manag..

[13] Wang Z., Zhang X., Jia S., Zhu Y., Chen L., Fu L. (2017). Influences of dynamic impregnating on morphologies and thermal properties of polyethylene glycol-based composite as shape-stabilized PCMs. J. Therm. Anal. Calorim..

[14] Karaman S., Karaipekli A., Sarı A., Bicer A. (2011). Polyethylene glycol (PEG)/diatomite composite as a novel form-stable phase change material for thermal energy storage. Sol. Energy Mater. Sol. Cells.

[15] Zalba B., Marin J.M., Cabeza L.F., Mehling H. (2003). Review on thermal energy storage with phase change: Materials, heat transfer analysis and applications. Appl. Therm. Eng..

[16] Li J.F., Lu W., Zeng Y.B., Luo Z.P. (2014). Simultaneous enhancement of latent heat and thermal conductivity of docosane-based phase change material in the presence of spongy graphene. Sol. Energy Mater. Sol. Cells.

[17] Sari A. (2015). Fabrication and thermal characterization of kaolin-based composite phase change materials for latent heat storage in buildings. Energy Build..

[18] Sari A., Alkan C., Bicer A., Bilgin C. (2014). Latent heat energy storage characteristics of building composites of bentonite clay and pumice sand with different organic PCMs. Int. J. Energy Res..

[19] Tang L.-S., Yang J., Bao R.-Y., Liu Z.-Y., Xie B.-H., Yang M.-B., Yang W. (2017). Polyethylene glycol/graphene oxide aerogel shape-stabilized phase change materials for photo-to-thermal energy conversion and storage via tuning the oxidation degree of graphene oxide. Energy Convers. Manag..

[20] Qian T., Li J., Feng W., Nian H.E. (2017). Single-walled carbon nanotube for shape stabilization and enhanced phase change heat transfer of polyethylene glycol phase change material. Energy Convers. Manag..

[21] Yang J., Zhang E., Li X., Zhang Y., Qu J., Yu Z.-Z. (2016). Cellulose/graphene aerogel supported phase change composites with high thermal conductivity and good shape stability for thermal energy storage. Carbon.

[22] Mehrali M., Latibari S.T., Mehrali M., Mahlia T.M.I., Metselaar H.S.C. (2013). Preparation and properties of highly conductive palmitic acid/graphene oxide composites as thermal energy storage materials. Energy.

[23] Patil M.P., Gaikwad N.J. (2011). Characterization of gliclazide-polyethylene glycol solid dispersion and its effect on dissolution. Braz. J. Pharm. Sci..

[24] Wang C., Feng L., Yang H., Xin G., Li W., Zheng J., Tian W., Li X. (2012). Graphene oxide stabilized polyethylene glycol for heat storage. Phys. Chem. Chem. Phys..

[25] Liang W., Chen P., Sun H., Zhu Z., Li A. (2014). Innovative spongy attapulgite loaded with n-carboxylic acids as composite phase change materials for thermal energy storage. RSC Adv..

[26] Qi G., Yang J., Bao R., Xia D., Cao M., Yang W., Yang M., Wei D. (2017). Hierarchical graphene foam-based phase change materials with enhanced thermal conductivity and shape stability for efficient solar-to-thermal energy conversion and storage. Nano Res..

[27] Yang J., Qi G.-Q., Liu Y., Bao R.-Y., Liu Z.-Y., Yang W., Xie B.-H., Yang M.-B. (2016). Hybrid graphene aerogels/phase change material composites: Thermal conductivity, shape-stabilization and light-to-thermal energy storage. Carbon.

